# Ethics of a Physiotherapist: Touch, Corporeality, Intimacy—Based on the Experience of Elderly Patients

**DOI:** 10.1007/s11673-023-10323-x

**Published:** 2024-05-15

**Authors:** A. Długołęcka, M. Jagodzińska, W. J. Bober, A. Przyłuska-Fiszer

**Affiliations:** Faculty of Rehabilitation, Józef Piłsudski University of Physical Education in Warsaw, Marymoncka 34, 00-968, 45 Warsaw, Poland

**Keywords:** Ethics of a physiotherapist, Touch, Corporeality, Intimacy, Grounded theory

## Abstract

This paper presents a qualitative study investigating the application of physiotherapists’ professional ethics in practice with respect to touch, intimacy, and corporeality during therapy, based on the experiences of elderly patients. As the relationship in a physiotherapy session is multidimensional, the study considered three levels: physical contact, verbal contact, and the conditions in which the therapy took place. The aim of this study was to find out what values are of importance to older people during a physiotherapy session, with emphasis on the categories of touch, corporeality, and intimacy. The studied group consisted of sixteen male and female physiotherapy patients aged between sixty-six and ninety-two years. The study was conducted according to the grounded theory methodology. The research material consisted of transcriptions of free targeted interviews, which were subjected to a process of coding and analysis. As a result of data analyses, three superior categories have been identified—safety, anxiety, interpersonal relationship—and three a priori categories stemming from the characteristic features of the study area—touch, corporeality, and intimacy. The a priori categories did not appear independently in statements made by the respondents, but instead seemed to be components of superior categories. The most important values indicated by the respondents concerned the interpersonal relationship with their physiotherapist and the feeling of safety and care. In terms of touch, corporeality, and intimacy, the respondents indicated, among others, the importance of predictability, a sense of security, privacy, and acceptance of the body.

## Introduction

According to the “Rules of Professional Ethics” adopted by the National Chamber of Physiotherapists (Krajowa Izba Fizjoterapeutów (KIF)) in Poland: “Physiotherapists are guided in their professional practice by the values of care, professionalism, responsibility, fairness, professional integrity as well as respect for dignity and autonomy of a patient” (KIF 2022, 3). This is an international standard—it is addressed at different levels of detail by, for example, the American “Code of Ethics for the Physical Therapist” (APTA 2020), the French “Code de déontologie des masseurs-kinésithérapeutes” (ORDREMK 2022), and the Maltese “Code of Ethical Conduct for Physiotherapists” (MAP 2017). This may be justified by the studies of Kulju et al. (2013) who conducted a qualitative research study on moral sensitivity and found out that physical therapists encounter ethical problems in their everyday practice and that unethical behaviour of physiotherapists and other health professionals is an important subcategory of these problems. Codes of professional conduct, therefore, provide support for people for whom ethical professional practice is important but who are not always able to make the judgement by themselves.

**Fig. 1:**
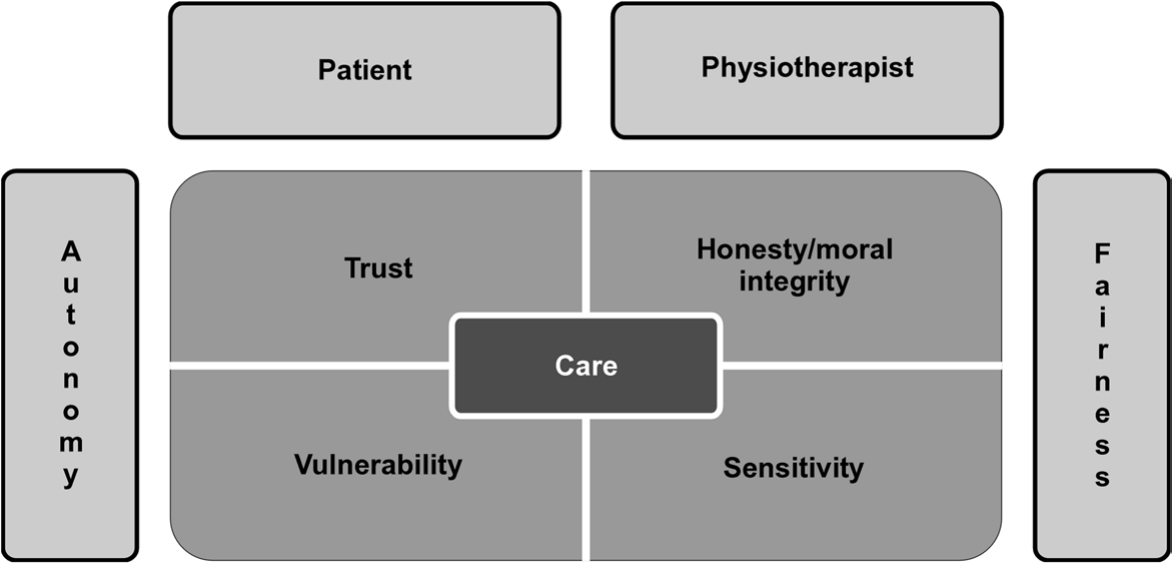
Values in a physiotherapeutic relation (Przyłuska-Fiszer and Wójcik, 2020, 130)

The scope of ethical issues in this paper is set in the context of the axiological model of a therapeutic relationship in physiotherapy (Figure 1) developed by Alicja Przyłuska-Fiszer and Agnieszka Wójcik (2020). It comprises two fields: (1) issues of the patient–therapist relationship: frameworks for professional behaviour; issues of communication, privacy, autonomy, and consensuality; and asymmetries between knowledge and power (Praestegaard and Gard 2013), and (2) issues of moral stance, with particular reference to the ethics of care.

## Touch, Corporeality, and Intimacy in Physiotherapy

Since the practice of physiotherapy emerged as a field based largely on manual work with the body, it has become problematic to find a formula in which touch and corporeality in therapy would not have sexual connotations. Physiotherapy is nowadays classified in the so-called bodywork category, where the body is, on the one hand, the physiotherapist’s working tool and, on the other, the object of work (Krzesicka 2019; Twigg et al. 2011). The issues of desexualisation of the body and the therapeutic relationship remain quite relevant and have been reflected in the biomedical approach, rooted in the Cartesian concept of the body-as-machine. In this way, the practice of physiotherapy came closer to the model of doctors’ work: the therapy was set on a couch either in a hospital environment or in a desensitised office, and physiotherapists donned uniforms—medical gowns (Gimlin 2007; Nicholls and Holmes 2012). Meanwhile, current philosophical discourse and the social sciences tend to consider embodiment to a much bigger extent from a phenomenological perspective, following Merleau-Ponty in recognizing the experience of the body as an essential element of perception and a way of being-in-the-world, and even “that the bodily-self constitutes the most fundamental form of the conscious mind” (Przybylski 2015, 55–56; Długołęcka 2019). And, consequently, the issues of embodiment and the relationship to one’s own body and to the body of another person are of integral importance to both parties involved in the physiotherapy encounter, albeit differing in their social roles.

While corporeality is an inseparable part of the sense of identity, touch, in Ratcliffe’s (2012) terms, determines body awareness. Touch carries the meanings given to it by the toucher and the touched person. It can be a means of expression of “giving”: care, tenderness, love, safety, presence; on the other hand, it can also express “taking”: desire, appropriation, domination, violence. In physiotherapy, touch is an indispensable diagnostic and therapeutic tool.

In the context of a physiotherapy session, two categories of touch may be distinguished: *therapeutic* touch, which includes diagnostic, intervention, accompanying, and informative types of touch, and *non-therapeutic* touch, which includes caring touch, relationship building, and preparation of the patient (Przyłuska-Fiszer and Wójcik 2020; Davin et al. 2019). On the other hand, in a meta-ethnographic study comprehensively covering the health professions (nursing, medicine, physiotherapy, osteopathy, counselling, psychotherapy, and dentistry) Kelly et al. (2018) hypothesized that touch in the therapeutic relationship can be: “(I) an expression of caring or (II) demonstration of power, and that it (III) requires safe space.” By investigating the importance of touch in particular fields, they have established that (in the opinion of patients or therapists) touch plays a therapeutic role in physiotherapeutic and osteopathic practice because it is a tool for communication and for showing care intended to establish a relationship, giving a sense of security (Kelly et al. 2018). Touch carries with it the meanings given to it by the person doing the touching and the one being touched, but the same act of touching can acquire different meanings for the two persons (and another meaning for the person who observes from the outside). By accepting touch, an internal calculation is made, on the basis of which the acceptability of the touch is assessed. As criteria for this calculation, Heslin and Alper (1982 as cited in Benjamin and Sohnen-Moe 2013, 134) specify the following: What part of the other person’s body is touched? What part of my body is touched? How long does the touch last? What is the strength of the touch? Was there any movement after the contact was made? Is anyone witnessing the touch and if so, who? What is the relationship between me and the person who is touching me? What situation did the touch take place in? What words accompany the touch? What non-verbal behaviour accompanies it? What are my past experiences with the person who is touching me?

One important area of analysis in this paper will be the importance that respondents ascribe to touch, the extent to which they distinguish between different modalities of the tactile relationship, and whether they have experiences (and which ones) of crossing boundaries within touch.

Intimacy, in general, means something of very personal nature. Edward T. Hall has defined intimate social distance, under which he included the distance of sexual activity, wrestling, protecting, and comforting (Hall 1990, 117–118). The category of intimacy can be interpreted much more broadly, as the hard-to-define privacy of inner life, probably best described by Mariola Bieńko (2013, 10):

The essence of intimacy is inaccessibility due to the subjectivity and uniqueness of experiences. It is a sphere of the most inner and often undisclosed sensations, quite difficult to express, an individual’s own secret history, a fragile social relationship that requires both a plan and a strategy, as well as discretion and tact to survive in its unique and exceptional nature. One could risk the thesis that it does not require a definition at all, as it is felt, practised and ultimately discursively inexpressible.

## Old Age and Ageism

Old age and ageing are embedded in the cultural context, and this will influence the relationship of the surroundings and the internal relationship of the older person to himself or herself (Ayalon and Tesch-Römer 2018). In this sense, old age is a social construct. Serra et al. (2011, followingKydd et al. 2018) have proposed that people should no longer be described as “old” or “young” but instead be categorized by the decade of their life (centenarians, octogenarians, etc.); this approach has not been widely adopted so far. The current gerontological discourse divides old age into sub-periods: Eurostat (2019) uses the term “older persons” for people over sixty-five years of age and “very old persons” for people over eighty-five years of age; divisions into “young-old,” “middle-old,” and “old-old” have been proposed as well (Kite and Wagner 2002).The term *ageism* was coined in 1969 by Robert N. Butler, who used it to describe discriminatory behaviour and attitudes towards people on the basis of their age, in particular towards the elderly (Butler 1969). Ayalon and Tesch-Römer (2018) derive ageism from generalizing and stereotyping the ageing process, which in fact has multiple and unique courses and is an effect of diverse and heterogeneous life situations.

Women and men are stereotypically attributed different qualities, and their old age is generalized differently in the same way; older women, for example, are attributed a warm personality, emotionality, and sensitivity to the feelings of others, while men are attributed self-confidence, assertiveness, and perseverance (Kite and Wagner 2002). In literature, this has been given its own specific name—*gendered ageism* (Kite and Wagner 2002; Krekula et al. 2018; Barrett and Naiman-Sessions 2016). In its negative implications, it can become another dimension of prejudice against the elderly, increasing their vulnerability.

Many forms of ageism have been found in healthcare, from problems at the systemic level, related to the allocation of resources and availability of specialists qualified in geriatrics, through lower quality of diagnostics and treatment, to worse treatment in direct contact such as time spent on care, verbal and non-verbal communication, and exclusion from the decision-making process (Wyman et al. 2018). Kropińska (2013, after Burak and Reczyńska 2015) finds that 14.9 per cent of older people have experienced and 19.4 per cent have witnessed discrimination against older people in their dealings with healthcare. Ageist attitudes have negative impact on the quality of care offered to older people (Giles et al. 2002). Some studies point to a divergence of beliefs and attitudes in physiotherapy students: on the one hand, it has been shown that most students have positive beliefs concerning older people (Inbar et al. 2012; Kalu, et al. 2018; Duthie and Donaghy 2009), but concurrently they are not willing to work with them or do not consider working with them. It also revealed the positive impact of contact with older people on reducing prejudice against them.

## Aim of the Study

The starting point is a model representing the values of physiotherapists and patients during a physiotherapy session (Przyłuska-Fiszer and Wójcik 2020). The aim of the research was to find out what values older people give importance to during a physiotherapy encounter. The following questions were asked during the study:

1. To what extent do values expressed by the patients in their descriptions of their experiences coincide with their expectations about an ideal physiotherapy encounter?
2. What, based on the experiences of elderly patients, is included in the categories of touch, corporeality, and intimacy in the context of a physiotherapy encounter?
3. What are the needs of elderly patients in terms of touch, corporeality, and intimacy in the context of a physiotherapy encounter?

## Methods

The study was carried out using the grounded theory methodology based on the assumptions of symbolic interactionism (Konecki 2000). Its distinguishing feature is the reordering of activities in the process of theory building. As Glaser and Strauss (1999) noted, it is a process in which empirical confirmation is sought for an abstract concept and is accompanied by a characteristic rhetoric, the so-called *rhetoric of verification*. In the grounded theory methodology, a study is carried out first (data compilation), then the gathered data are handled in a coding process to derive hypotheses and to generate a theory based on the observed regularities. In such a way, theories are “grounded empirically,” that is, in facts (Glaser and Strauss 1999). The coding process is an intermediate step in the construction of a grounded theory. It begins by assigning as many categories (or codes) as possible to each event which emerge in the course of analysing the material; some categories may be predetermined, depending on the researcher’s area of information needs (Konecki 2000).

## Study Group and Course of the Survey

The survey group consisted of sixteen elderly people (eight women and eight men) from Poland who had experience in participating in physiotherapy as patients. The patients had experience with many physiotherapists and, therefore, the gender of physiotherapists was not taken into account in the study. For the purpose of this study, sixty-five years of age was assumed as the lower age limit, based on the classification of Eurostat (2019). The average age of respondents is 73.8 years (SD=8.1). The material was compiled according to the methodology of a free targeted interview. In the course of this research, a list of issues was used, which was divided into two parts: the descriptive part (questions about the respondent’s experiences) and the normative part (what, based on experiences, should a physiotherapy encounter be like).

The coding process used the methodology of Kathy Charmaz (2006, 42–72), according to which data processing progresses in several iterations—coding the data several times, at different levels of generality. The process was divided into three stages: *initial coding,* then *focused coding*, and finally *theoretical coding*. In the phase of *theoretical coding*, building on the basis of the two preceding steps, groups of codes (referred to by Charmaz as “code families”) were identified and ascribed to the most general categories. This is the most synthetic stage, which makes it possible to observe relationships and, further on, to build theory. In the course of coding and analyses of compiled data, certain motives emerged which were subsumed under a number of subcategories from which the following superior categories were identified: security, anxiety, and interpersonal relationship. In order to structure the relationships between them in the workflow, a mind-map was prepared and used to create simplified models, as presented in the following sections.

In addition to the categories identified during data analysis, three a priori categories were developed separately, determined on the basis of characteristics of the study area: touch, corporeality, and intimacy.

## A Priori Category: *Touch*

The category of touch rarely emerged spontaneously in statements made by the respondents—but usually as a result of the moderator directly asking them to distinguish between professional and non-professional touch or in conjunction with issues of consent or boundary crossing. In terms of such subcategories, touch is presented below.

For all respondents, the distinction between professional and non-professional touch appeared to come down to the question of its “necessity,” with some people referring to therapeutic touch in this way, while some still noted accompanying touch; nevertheless, both categories fall within the boundaries of therapeutic touch. It seems that some respondents understood intentionally going beyond the therapeutic aims as unnecessary touch. Acting as “professional touchers,” physiotherapists may feel that physical contact with patients is something so obvious that they do not necessarily even take note of it; however, this feeling is not symmetrical, and there may be discomfort on the part of the patient, even if the touch does not carry any intention. Although affective touch does not fall within the bounds of professionalism as defined by the respondents, they had a nuanced attitude to it, but one that was consistent with their general approach to proximity (beyond the physiotherapy encounter). And so, one female respondent, who described herself as needing more distance, did not accept affective touch in the office. Another one, on the contrary, declared that touch contact is natural for her and that she happened to hug with her physiotherapist.

Four of the respondents expressed the expectation that the physiotherapist would demonstrate interpersonal skills of a high enough level (“Needs to be a psychologist”) to be able to intuitively understand the patient’s needs in this area, especially needs of older patients.

In the interviews concerning touch, it was notable that the interviewees did not notice any variation in the qualitative characteristics of touch and ways of touching. The only exceptions were the aforementioned affective touch as well as sexual touch; beyond that, the respondents had few observations. It has been noted that different people have different touch, but modalities such as hand temperature, pressure, type of grip, and others seem to remain beyond the awareness of the interviewees.

Most people did not experience bodily boundary crossing; however, two respondents experienced abuse and one experienced a physiotherapist error of touching with too much force. In the theoretical context, when talking hypothetically about boundary crossings, respondents mentioned sexual harassment as the only violation and had a noticeable difficulty in expressing their thoughts: some people described the abuse as unimaginable, while others started to use special phrases that allowed them to avoid expressing themselves directly. Two of the interviewees rated the physiotherapy encounter as the worst they had ever experienced, precisely because of the touch that crossed boundaries. The feeling of the impunity of physiotherapists is a topic that came up several times, and for that reason it will be mentioned again in the categories of anxiety and helplessness.

The issue of asking for consent to apply touch during therapy was raised in every interview. Only one respondent stated that the physiotherapist should ask for consent before starting the therapy, and that was exactly what he had experienced. Moreover, the respondents presented different attitudes: two interviewees stated that such a question was unnecessary (however, during the interview, both of them added that this applied only in the case of men—women should be asked); the remaining persons expressed only the need to be informed about the planned procedures before the treatment is actually started. Such information has two functions: it either allows psychological preparation (gives a sense of security) or creates space for refusal.

The conclusion that emerges from the interviews is that prior to the interviews, the concept of consent was unknown to this group of respondents; for some of them it remains incomprehensible. It seems that for some of the interviewees, the very fact of coming to get a treatment implies implicit consent to all procedures that the therapist deems necessary.

## A Priori Category: *Intimacy*

In the spontaneous course of comments, the concept of intimacy appeared infrequently, usually in the following two senses: (1) privacy and (2) as a term for genitals or erogenous zones of the body. When asked directly about their understanding of intimacy, the respondents had a (fully understood) difficulty in defining this concept, but usually the answer tended to hover around sexual relations. The interviewees were also asked to state their opinion on the acceptability of close relationships between patients and physiotherapists. The interviewees used the term intimacy when addressing not being exposed during therapy to third parties: either other patients or staff. The interviewees took into account not only their own sense of intimacy, but also the intimacy of other patients, which they did not want to violate. Privacy understood in this way was linked to the category of shame, corporeality, and revealing the body (nudity). Intimacy was connected to corporeality also through speech: the respondents were sensitive to the way they talked about their bodies, especially if it concerned issues they found important (particularly obesity); in this sense, the physiotherapist could verbally hurt the patient quite deeply. In a broader context, intimacy was important for building a sense of security or causing a sense of anxiety.

The second understanding of intimacy as privacy concerned the communicative sphere, that is, conversations concerning health issues or elements of private life unrelated to therapy. Communicative intimacy could concern the respondent and his therapist but also pertained to audible conversations between other physiotherapists or other patients and their therapists.

Asked to describe what intimacy is, the respondents adopted different strategies. The majority of them described situations in which intimacy reveals itself in a significant way. One interviewee told the story of a dramatic romance from a soap opera. Only two respondents attempted to approach the problem in a more personalized way, trying to describe what *their* intimacy actually is. Two understandings emerge from these definitions. The first is of a type of close relationship that may be sexual in origin or is exclusively sexual. In the second approach, intimacy was defined as a sphere of closeness including a bodily and psychological aspect. Other people are deliberately allowed—or not—into this sphere. Some respondents express discrepancy of meaning in the way they use the word “intimacy” in the spontaneous course of speech and when trying to conceptualize this notion. For example, in one interview, a respondent spontaneously used “intimacy” in the sense of “privacy”; when asked for a definition, he defined intimacy as a close relationship of a sexual nature. It seems that the conceptual scope of intimacy is much broader than respondents can express in words. In the theoretical part of the study, the respondents were asked about close relationships between patients and physiotherapists and whether it is acceptable to have a friendship, flirt, or romance. All of them found such forms of relationships acceptable provided they were consensual, most often with the proviso that showing closeness should not take place during therapy as long as it takes place in the office. In a private setting, during home therapy they allowed interactions that went beyond professionalism.

## A Priori Category: *Corporeality*

Corporeality in the respondents’ statements is set in the context of their relationship to their own bodies, as well as relationship of the physiotherapists, and is presented in relation to the category of shame. The second context is the category of old age, linked to categories of fitness, appearance, and vulnerability. Embarrassment during a physiotherapy appointment was the most common topic at the occasion of which the respondents started talking about the body. According to them, embarrassment may appear in patients when it is necessary to uncover their body: on the one hand because of the physiotherapist’s gaze intruding into an intimate sphere, but mainly because of the belief that one’s own body does not conform to the norms. And so, negligence of hygiene may be a reason for shame, but also visible signs of old age, changes that arise from illness, scars, or simply failure to conform to internalized beauty canons. Only one interviewee indicated the behaviour of the therapist consisting in commenting on the appearance of the body or its reactions or the way the patient performs movements as a possible source of embarrassment. The remaining respondents mentioned low self-esteem and fear of being looked at critically as a factor, thus placing the responsibility for the feeling of embarrassment on the patient. At the same time, all participants declared that they had never experienced the feeling of embarrassment during a physiotherapy appointment. Some people took conscious steps to prevent such discomfort, by taking up special hygienic measures on the day of treatment or choosing new underwear.

It was noticeable that when describing the body (their own, that of other patients, or of hypothetical people), male and female participants only mentioned undesirable features, or used pejorative terms. None of the people spoke about what they liked about their body, and instead focused on criticism. The terms that were used were “ugliness,” “deformity,” “negativity,” “fat” or (we read in one interview): “Well, because there are shapely girls, and shapely women, and those like me. I dislike my breasts, my terribly big breasts.” In the case of one person, there was a statement indicating a kind of dissociation or externalization of the body. Concurrently, two of the respondents observed a certain social change towards better self-acceptance in women, a decrease in the sense of embarrassment with age, and more freedom to uncover the body. The women interviewed pointed to several events that were helpful for them in building an accepting relationship to their own corporeality: positive motivation from a young physiotherapist running classes for women at the swimming pool or an encounter with the diversity of women’s bodies in the swimming pool locker room.

## Superior Category: *Security*

The sense of security can be understood as one of the extremes on the axis of the spectrum of *anxiety ↔ security*, where the lower the level of anxiety, the greater the sense of security, and vice versa. However, from the respondents’ statements obtained in this study, it appears to be a more nuanced phenomenon: what emerges from the interviews is a picture of feeling safe as an internal state that is positive in the sense that there is more behind it than just a lack of anxiety. In this approach, the sense of anxiety and the sense of security can be visualized on two separate axes. Later on in this paper, the factors that increase the value on the security axis will be presented (subcategories: sense of care, features of the physiotherapist, predictability, therapy conditions) and then those that have an impact on the feeling of anxiety of older people during a physiotherapeutic session.

Respondents mentioned “feeling cared for” as a positive differentiator when describing their positive experiences and, in the theoretical part of the interview, as a need that should be met, particularly in older people. The category of feeling cared for also includes statements pointing to the need for care, involvement, and individualization of therapy. The conducted interviews indicate that the feeling of being taken care of was satisfied when the respondent felt they were the focus of the physiotherapist’s attention both in terms of the delivery of therapy and of interpersonal relationship. In the context of conducting therapy, this meant listening to the patient, interviewing them (also in the case of a medical recommendation), and asking for feedback—and consequently adapting actions to current needs. In particular, respondents who participated in rehabilitation implemented under the National Health Fund paid particular attention to the individualization of therapy. Taking care also included noticing the patients’ needs resulting from, among other things, age-related limitations in mobility, and creating comfortable conditions.

The sense of security as reported by the interviewees was influenced by certain qualities of the therapists that made them trustworthy. When asked about the criteria for choosing a physiotherapist, the respondents most frequently indicated competence and effectiveness. The belief in competence was based on experience and, in the case of the first contact, on the *impression* of competence. Apart from the belief that the physiotherapist would be effective, of importance were the qualities related to the way of being and functioning in the consulting room: confidence, calmness, and warm contact.

The need for predictability of the physiotherapy encounter was expressed mainly in aspects related to full information and good communication. The first aspect is related to the category of consent to touch described earlier on: the respondents indicate the necessity of anticipating and describing the procedure before any action could be commenced—reserving space to express objections, but also to mentally brace themselves. Good communication also includes information on how to become prepared for the procedure (inadequate preparation was mentioned as a possible cause for embarrassment) and an appropriate outline of the meeting at the outset.

Another element that builds the respondents’ sense of security consisted of conditions in which the therapy was taking place—primarily in relation to the categories of intimacy and corporeality described earlier on, and consequently to maintaining privacy in situations where the patient had to expose his or her body. The need for being isolated from the others varied in degree among the respondents; for example, separating the workstations with curtains was sufficient for some, while others also needed to ensure the privacy of their conversations or to be isolated from ambient sounds that made it difficult to concentrate. Some female respondents pointed to the inner feeling of certainty that no person would enter the room. From the experiences described, it appears that the physiotherapists paid attention to maintaining the patients’ privacy by leaving the room while they were preparing for the procedure—such behaviour was noticed and appreciated by female respondents.

## Superior Category: *Anxiety*

During the conducted interviews the respondents did not use terms that explicitly described anxiety, apprehension, or fear; the category of anxiety emerged during the analysis of codes from the focused coding stage and includes some physiotherapist behaviours, therapy circumstances, and respondents’ beliefs about themselves. Phenomena that affect feelings of anxiety are grouped into subcategories: vulnerability, error in therapy, embarrassment, abuse, and uncertainty. Respondents presented the physiotherapy encounter as a hierarchical relationship in which their position was lower. This was due, on the one hand, to the fact that they were the ones coming for help (one person used the term “supplicant”) and, on the other hand, to the difference in knowledge: it is the physiotherapist who decides on the course of the visit, knows what should be done, understands the patient’s body and health better than they do. Foucault’s asymmetry of knowledge and power put them in a position of vulnerability. The feeling of vulnerability was also intensified by symptoms of ageing: reduced dexterity that requires special care from the therapist, memory problems, and fragility of the body, which may be easily injured by inadequate therapy.

Another aspect of vulnerability stems from the beliefs concerning corporeality that have already been described. When asked about boundary-crossing behaviours, all respondents indicated commenting on the patient’s body by the physiotherapist. This seems to be a particular category: by comparison, while for some of the interviewees (particularly men) the prospect of crossing the boundaries of touch was not a realistic scenario, commenting on the body is a boundary violation that could befall anyone. One male respondent received a positive comment, expressing appreciation of his fitness. In contrast, verbal abuse was experienced only by women among all respondents. Obesity was a particularly sensitive issue—some people defined their body as obese, and comments about it were particularly hurtful.

In the case of several respondents, physiotherapists made mistakes that caused pain or health impairment and, as a result, caused increased caution or anxiety, due to which the interviewees happened to abandon the therapy. Physiotherapists who made a mistake or committed malpractice suffered no consequences in any of the situations described. When aggrieved respondents attempted to clarify the issue with their therapist, they were not always successful, nor did all interviewees feel able to take on a confrontation, nor did any of them decided to proceed with the case formally. It can be assumed that the impunity of physiotherapists contributes to the feeling of powerlessness on the part of patients and their anticipation of similar situations during subsequent treatments.

From the descriptions of the respondents’ experiences, a picture emerges in which patients were kept in uncertainty at several levels during the therapeutic visit. On the therapeutic side, they were not informed about the course of the visit, and they were not informed of the type of treatment, goals, or prognosis of the therapy. Meanwhile, from the theoretical part of the interviews, it is known that respondents have a need for information and predictability.

Uncertainty also emerged in the systemic context, where the most frequently mentioned issues were those related to time management such as punctuality for start of the treatment and respecting the allocated treatment time: respondents encountered both shortening and over-extending the treatment duration. As a result, interviewees having several treatments in a row were not able to predict how much time they would spend at the rehabilitation facility. One person encountered a situation where the agreed therapist was replaced by another one without prior notice—the person found that unacceptable. It should be added that one of the systemic problems related to uncertainty was insufficient access to rehabilitation; two respondents resorted to black humour by stating with amusement that they might not live to see the dates on which they have their next therapy planned.

## Superior Category: *Interpersonal Relationship*

The interpersonal relationship is the last of the superior categories described in this paper; nevertheless, in their statements the interviewees ascribed the highest importance to its various aspects. The issues of personalized relationship raised by the respondents were reduced to sub-categories: subjectivity, caring, conversation, features of physiotherapists and patients. The need to be the focus of the physiotherapist’s attention was marked in the respondents’ statements. This applied both to the way the therapy was conducted (in an individualized way, taking into account the interview, providing feedback, and responding to current needs), but also to the feeling of being treated as a full person, or the need to be *noticed*. However, the experiences of the interviewees show some neglect of the relationship on the part of the physiotherapists, which comprised impersonal treatment, ignoring the presence, or working as if “in spite of” the patients. Private conversations between physiotherapists and/or using the telephone during therapy were particularly negatively perceived; as a result, respondents had a sense of being objectified, of being a spare, unimportant part of therapy.

The notion of caring as such did not appear frequently in the interviews but had been recognized as a category on the basis of a range of needs and opinions expressed indirectly by the respondents. As such, caring is indeed linked to the previously described sense of security and, in particular, to a sense of being taken care of and to subjectivity.

Caring, according to the respondents, creates a working atmosphere in which they feel comfortable in a physical sense, by ensuring their comfort and safety, and in a psychological sense, by physiotherapists adapting the way they communicate with the patient and taking into account their emotional state. Nevertheless, the need for caring has not always been met. Respondents found themselves in situations where they were left to themselves, causing them to feel anxious as a result, and in one case this even led to a decline in their condition. In a similar way to the need for empowerment, neglect in assuring care was often associated with situations when the therapist would talk on the phone during therapy.

All the respondents emphasized the importance of good contact with the physiotherapist; several of them considered it to be the most important criterion for evaluating a therapeutic encounter. Good communication most often depended on the qualities of the physiotherapist. From the more superficial qualities, the respondents mentioned politeness, cheerfulness, respect, contactability; moreover, higher interpersonal skills were expected: listening, empathy, and sense of distance, as well as relationship management and assertiveness skills. Two female respondents emphasized the importance of the voice and the way of speaking— for one of them this was a key feature. Some respondents distributed the responsibility for good contact on both sides of the relationship, indicating that the patient should also display respect and politeness. However, when describing patient characteristics, interviewees more often focused on undesirable characteristics: entitledness, demanding to “be served,” commenting. One of the interviewees shifted the entire burden of responsibility for good contact onto the patient and, in terms of negative characteristics, listed entitledness first and foremost, which he linked to old age.

Respondents often mentioned the need for a conversation during therapy—not necessarily related to the treatment itself. For them conversation tended to fulfil several functions: it responded to emotional needs and was a manifestation of interest (and, therefore, linked to categories of caring and subjectivity), building a more personalised relationship and filling the time. Some used the conversation as an opportunity to educate themselves in matters related to health. The subjects usually had extensive experience with rehabilitation, and their contacts with physiotherapists extended over many months—or even years. As a result, their relationships became closer and more personal and so did their conversations. After many sessions with one therapist, even people who considered themselves as reserved were willing to share intimate events from their lives—such as illness or the death of loved ones. This relationship of closeness in conversation was mutual—the physiotherapists also shared stories about their private lives. Nevertheless, the respondents pointed out that it should be up to the patient to make the relationship closer; flippancy and jokes were particularly unwelcome.

## Summary and Discussion

In the first place, it is important to emphasize the interviewees’ very positive attitudes towards physiotherapy and physiotherapists as such. Some interviewees had built such a positive relationship with their therapists (or were feeling real gratitude) that talking about even the slightest hypothetical abnormalities made them quite uncomfortable, or perhaps even made them feel disloyal. For this reason, the perception of a physiotherapy appointment presented by several respondents may be incomplete. While analysing results obtained during the survey, a certain structure of categories and relations between them has been identified. This structure is based on values that had been identified from the needs expressed by the respondents towards an ideal physiotherapy appointment and their experiences during real-life appointments.

**Fig. 2.**
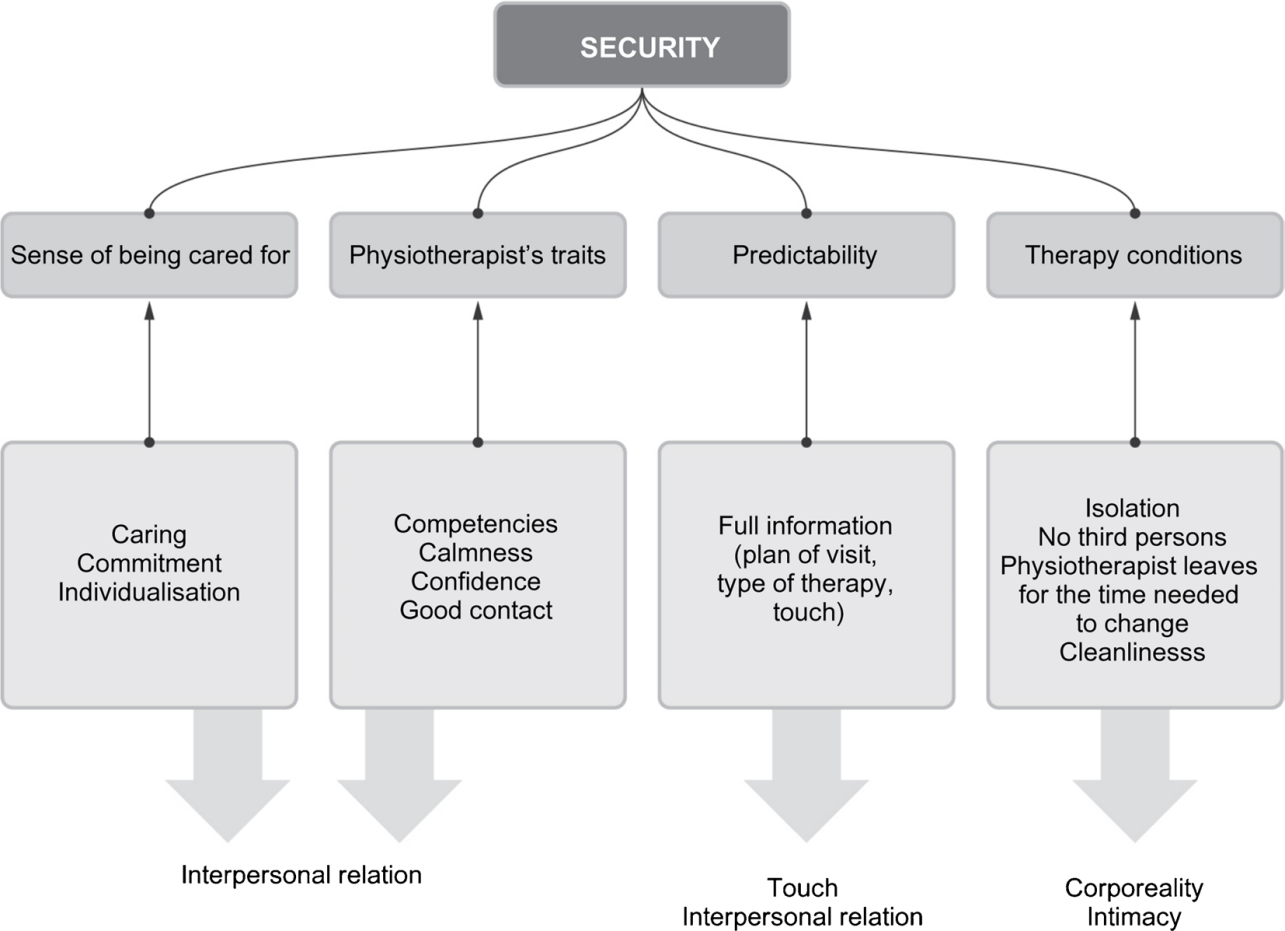
Simplified scheme of the security category [own study]

**Fig. 3.**
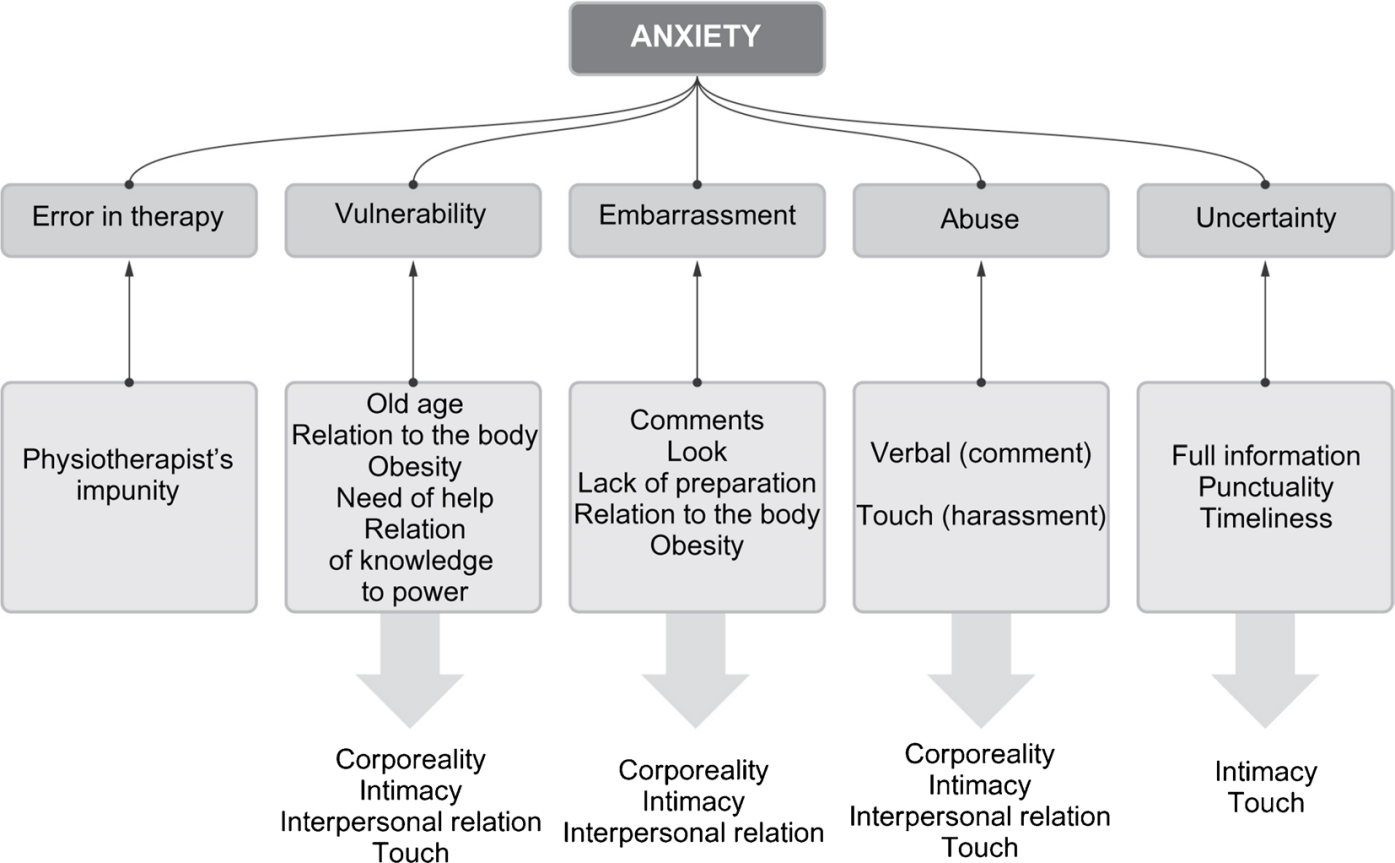
Simplified diagram of the anxiety category [own study]

**Fig. 4.**
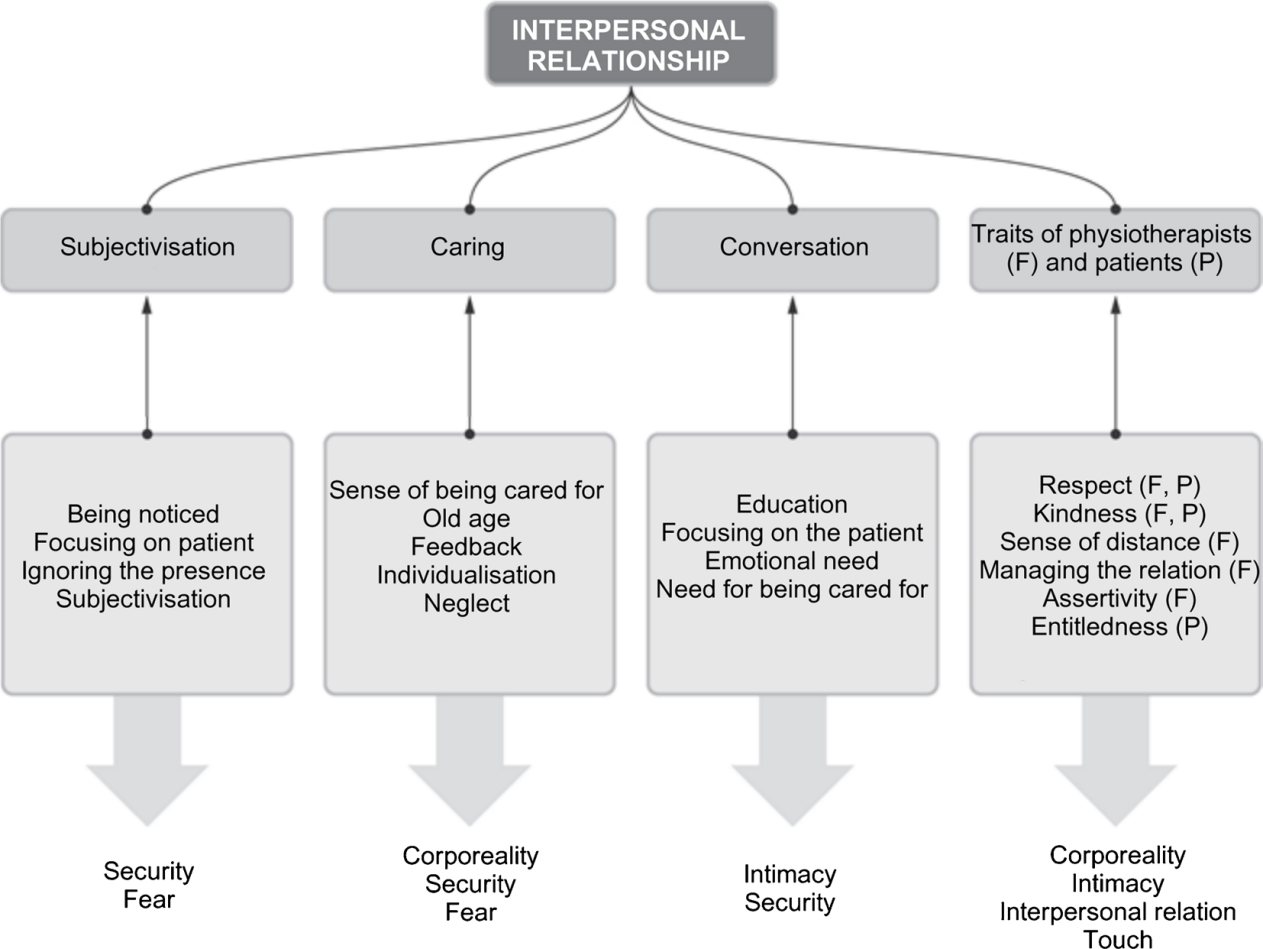
Simplified diagram of the interpersonal relationship category [own study]

The superior categories that emerged from the survey were security (Figure 2), anxiety (Figure 3), and interpersonal relationship (Figure 4). The subcategories and a priori categories that form them can belong to one or more superior categories, or function independently. This means that the relationship between categories can be both hierarchical and parallel.

Clearly the a priori categories were not given the highest weight in the respondents’ statements, despite their being asked about them in an explicit manner. Nevertheless, it is also noticeable that they were an important part of the superior categories—in particular, intimacy and corporeality. In the statements of female and male participants, it can be noted that issues related to touch were treated superficially. Despite in-depth questions, only therapeutic, accompanying, and sexual types of touch were distinguished in the scope of physiotherapy encounters, usually reduced to two categories: necessary and unnecessary touch (or professional and non-professional). It seems that such an approach is close to a mechanistic one and much narrower than the variety described by researchers of this subject (Davin et al. 2019; Kelly et al. 2018; Przyłuska-Fiszer and Wójcik 2020).

The broadest context of this study is the physiotherapist’s ethics; however, the experiences described by the participants indicate that the work ethos of the physiotherapist is also important: ensuring punctuality and treatment time, compiling patient history and feedback, tailoring treatment to patients’ needs, providing information about planned activities, and commitment to work.

The obtained results are compatible with the axiological model of a therapeutic relationship proposed by Przyłuska-Fiszer and Wójcik (2020), but with some differences. In particular, the categories of caring and vulnerability were crucial for the emergence of superior categories. On the other hand, the absence of certain issues in respondents’ interviews seems significant. The above-described model identifies patient autonomy as one of the limits for the therapist, whereas the codes of conduct for the medical professions (for example, the already mentioned codes of ethics of APTA, ORDREMK and MAP) also include respect for dignity, which is present in this model, even if not explicitly, but in the importance it attaches to trust, sensibility, and vulnerability. However, although the topics of corporeality, touch and intimacy, boundaries in therapy, and consent appear to have provided enough space to raise these issues, the participants of the present study did not mention them either explicitly or implicitly. The questions of consent and participation in therapeutic decision-making were not expected; for some respondents, simply coming to an appointment was a form of a priori consent to all therapeutic activities to be undertaken by the therapist.

None of the male or female interviewees described an event that would indicate ageist bias on the part of physiotherapists. On the other hand, some statements indicate ageist attitudes of the interviewees themselves on an affective and cognitive level (Kite and Wagner 2002); these manifested themselves, among other things, in a discourse about the body, but also in the attribution to older people of entitledness, inappropriate motivation, and unrealistic expectations of therapy.

## Conclusions

The survey allowed answers to be found to the following research questions:

1. To what extent do values expressed by the patients in descriptions of their experiences coincide with the model physiotherapy encounter they present?

None of the interviewees described a model physiotherapy encounter; however, some values were indicated against which the encounter was evaluated, or a criterion for choosing a physiotherapist was established. For some interviewees, these values changed during the course of the interview. And so, at the beginning of the interviews, six respondents mentioned competence and effectiveness of therapy as being the most important in their opinion, two mentioned availability of treatments, one mentioned gender, and three mentioned a good relationship with the therapist (while some people mentioned several values). In turn, at the end of the interview, the following were mentioned as most important: interpersonal relation (three persons), caring (two persons), subjective treatment (two people), and feeling safe (two people). One interviewee remained consistent in her opinion.

[2] What, based on the experiences of elderly patients, is included in the categories of touch, corporeality, and intimacy in the context of a physiotherapy encounter?

The category of touch, in the experience of study participants, includes the differentiation of touch into caring touch and accompanying touch, and abuse in the form of sexual harassment.

The category of intimacy comprises issues related to maintaining privacy (conditions in the consulting room), interpersonal contact (in particular, conversation), touch (massage as an “intimate matter”) and corporeality, in the context of uncovering the body.

An older person in a physiotherapy encounter reveals his/her vulnerability in many dimensions: he/she comes with a health problem, demonstrates some form of weakness, exposes his/her body, struggles with embarrassment, is touched. For this reason, the physiotherapy relationship as such can be considered intimate.

The category of corporeality concerned the relationship with one’s own body (expressed in the form of criticism) and embarrassment about one’s appearance, dysfunction, neglect, limited fitness, and visible aging processes. In this sense, it was closely linked to the category of intimacy. Moreover, the category of corporeality included vulnerability—both physical (due to the therapist’s mistake or abuse) and verbal (unwelcome comments).

[3] What are the needs of elderly patients regarding touch, corporeality, and intimacy in the context of a physiotherapy encounter?

The needs for touch expressed by the respondents oscillated around two issues: professionalism and predictability. Professional touch was defined as a therapeutically necessary touch, and therefore therapeutic or accompanying in nature. Predictability was associated with consent to touch. According to the interviewees, asking for consent is not required (and may even be unwelcome). Such opinions are in discordance with ethical and legal standards of informing patients that provide space for refusal.

In terms of intimacy, respondents expressed the need to feel safe and maintain their privacy when exposing their bodies and discussing their health.

In terms of corporeality, respondents were looking for a sense of security in accepting the imperfections of their body (in particular, not expressing any comments on its appearance) and adapting therapeutic activities to the needs and abilities of an older person.
